# 2bRAD-M reveals the difference in microbial distribution between cancerous and benign ovarian tissues

**DOI:** 10.3389/fmicb.2023.1231354

**Published:** 2023-08-24

**Authors:** Xiaogang Wang, Yaojun Zheng, Xiang Chen, Chen Peng, Shizhen Zhou, Sunan Shen, Shuli Zhao, Tingting Wang

**Affiliations:** ^1^The State Key Laboratory of Pharmaceutical Biotechnology, Division of Immunology, Medical School, Nanjing University, Nanjing, China; ^2^Jiangsu Key Laboratory of Molecular Medicine, Division of Immunology, Medical School, Nanjing University, Nanjing, China; ^3^General Clinical Research Center, Nanjing First Hospital, Nanjing Medical University, Nanjing, China

**Keywords:** ovarian cancer, microbiome, tumor microbial, 2bRAD-M, bacteria

## Abstract

The development of ovarian cancer is closely related to various factors, such as environmental, genetic and microbiological factors. In previous research, bacteria were identified in human tumors by 16S rRNA sequencing. However, the microbial biomass in tumor tissue is too low and cannot be accurately identified by 16S rRNA sequencing. In our study, we employ 2bRAD sequencing for Microbiome (2bRAD-M), a new sequencing technology capable of accurately characterizing the low biomass microbiome (bacteria, fungi and archaea) at species resolution. Here we surveyed 20 ovarian samples, including 10 ovarian cancer samples and 10 benign ovarian samples. The sequencing results showed that a total of 373 microbial species were identified in both two groups, of which 90 species shared in the two groups. The Meta statistic indicated that *Chlamydophila_abortus* and *CAG-873_sp900550395* were increased in the ovarian cancer tissues, while *Lawsonella_clevelandensis_A*, *Ralstonia_sp001078575*, *Brevundimonas_aurantiaca*, *Ralstonia_sp900115545*, *Ralstonia_pickettii*, *Corynebacterium_kefirresidentii*, *Corynebacterium_sp000478175*, *Brevibacillus_D_fluminis*, *Ralstonia_sp000620465*, and *Ralstonia_mannitolilytica* were more abundant in the benign ovarian tissues. This is the first use of 2bRAD-M technique to provide an important hint for better understanding of the ovarian cancer microbiome.

## 1. Introduction

Ovarian cancer represents one of the most challenging and lethal gynecologic cancers, and most patients have spread by the time of diagnosis (Carlson et al., 1994; Kossaï et al., 2018). Although ovarian cancer has the lowest incidence compared with other gynecological malignancies, it has the highest mortality rate (Coburn et al., 2017). Ovarian cancer is influenced by several factors, among which the presence of tumor microorganisms may have an impact on the development of ovarian cancer (Zhou et al., 2019). With the development of microbiome sequencing technology in recent years, it has been confirmed that microbes are indeed present in tumor tissues. Nejman *et al* examined the microbiome of seven tumor types (including breast, lung, ovarian, pancreatic, melanoma, bone, and brain cancers) by 5R 16S rRNA sequencing and found that each tumor type had a different microbial diversity (Nejman et al., 2020). Furthermore, research had shown that intracellular microbes in breast tumor cells could promote the metastasis of breast cancer (Fu et al., 2022). The colonization of *Fusobacterium nucleatum* in tumors could accelerate the progression of breast and colon cancer (Yu et al., 2017; Parhi et al., 2020; Chen et al., 2022). Obviously, tumor microbes play a crucial role in the development and metastasis of cancer.

The female reproductive tract contains a site-specific microbiome. Previously, it was believed that most bacteria were present in the lower female reproductive tract (including vagina and cervix), while the upper female reproductive tract (including the endometrium, ovary, and fallopian tube) was absolutely sterile (Ravel et al., 2011; Łaniewski et al., 2020). Recently, research had confirmed the presence of bacteria in ovarian cancer tissues by 5R 16S rRNA and 16S rRNA sequencing (Zhou et al., 2019; Nejman et al., 2020), and the distribution of bacteria in ovarian cancer and noncancerous ovarian tissues was different (Wang et al., 2020). Zhou et al. (2019) found that ovarian cancer microbes may influence the development and progression of ovarian cancer *via* modulating the local immune microenvironment. Local bacterial infections in the peritoneum as well as vaginal infections (such as *Neisseria gonorrhoeae* or *Chlamydia trachomatis*) may promote the development and metastasis of ovarian cancer, which may be caused by increased oxidative stress caused by inflammation and the resulting accumulation of DNA damage and mutations (Sipos et al., 2021). Additionally, pattern recognition receptors TLR2, 4, and 5 respond to bacteria or LPS and play a key role in ovarian cancer inflammatory drive (Muccioli and Benencia, 2014; Rutkowski et al., 2015; Kashani et al., 2020; Wang et al., 2020). Interestingly, not only was there a significant difference in the microbial composition between ovarian and benign ovarian patients, but also the microbiota in different parts of the reproductive tract in ovarian cancer patients was different (Brewster et al., 2022).

Presently, all relevant studies related to the ovarian cancer microbiome have used 16S rRNA sequencing. Since 16S rRNA sequencing only detects bacteria, and its sensitivity and resolution are low, generally only genus-level classification can be performed. And it is difficult to detect microbes at the species level. Furthermore, whole metagenome shotgun sequencing can also be performed for microbiome detection, but it is costly and requires a large initial biomass. Therefore, we attempted to apply a low-cost and high-resolution approach to characterize the low biomass ovarian microbiome at the species level. 2bRAD-M, a novel microbial sequencing technology, not only identifies bacteria at species resolution at low biomass, but also detects fungi and archaea (Lam et al., 2022; Sun et al., 2022). Moreover, this method can also detect high host contamination (tissue and blood, etc.) and severely degraded samples.

In the present study, we enrolled 10 patients with ovarian cysts and 10 patients with ovarian cancer. We performed the 2bRAD-M assay to analyze the microbial composition of both cancerous and non-cancerous ovaries and whether there were differences between the two groups. This is the first time that the 2bRAD-M approach has been used to analyze the microbiome composition of ovarian cancer at the species level.

## 2. Materials and methods

### 2.1. Sample collection

10 ovarian cancer (group C) and 10 benign ovarian (group B) tissues were all collected from Nanjing First Hospital. To minimize extrinsic factors that may affect the ovarian microbiome, ovarian tissue samples were placed in sterile freeze-storage tubes with sterile forceps and placed in liquid nitrogen without touching anything else. Inclusion criteria for patients in the group C: patients were initially diagnosed with suspected ovarian cancer followed by surgical resection, and the pathological findings confirmed ovarian cancer. Inclusion criteria for patients in the group B: patients were initially diagnosed with an ovarian cyst and underwent surgical resection, which was pathologically confirmed as a benign cyst. In addition, we also set the exclusion criteria for patient samples collection: treatment with antibiotics within 2 months, patients with chemotherapy, and patients with inflammation. Patient sample collection was approved by the Ethical Review Committee of Nanjing First Hospital. The samples were collected and used with the informed consent of the patients. The patients’ clinical information was collected and listed in Supplementary Table S1.

### 2.2. DNA extraction, library construction and sequencing

The tissues DNA was extracted by a TIANamp Micro DNA Kit following the manufacturer’s instructions (Tiangen, China). The 2bRAD-M library preparation followed the previous research (Wang et al., 2012; Sun et al., 2022). Briefly, the DNA (≤200 ng) was digested using BcgI restriction enzyme (NEB, America) at 37°C for 3 hours. Then, the above digestion products were ligated to the adaptors (Ada1, Ada2) overnight at 4°C using T4 DNA ligase (NEB, America). Subsequently, the above ligation products were amplified by PCR using Phusion High Fidelity DNA polymerase (NEB, America). The obtained PCR products were purified with QIAquick PCR purification kit (Qiagen, CA) and then sequenced with Illumina Nova PE150 platform. 2bRAD-M sequencing was performed at Qingdao OE Biotechnology Co., LTD. All adaptors and primers are listed in Supplementary Table S2.

### 2.3. 2bRAD microbial database construction

First of all, 173,165 microbial genomes (including bacterial, fungal and archaeal) from the NCBI RefSeq database were obtained. Next, all genomes obtained were electronically cleaved using 16 type 2b restriction enzyme. Ultimately, the acquisition of a specific taxonomic unit (no overlap with other species under that taxonomic unit) as species-specific 2bRAD markers, generating 2bRAD microbial genome database.

### 2.4. Relative abundance calculation

Firstly, mapping of all sequenced 2bRAD tags after quality control (QC) to the constructed 2bRAD marker database. In order to control false positives, the G score was calculated for each species using the formula shown below: G score _species i_ = $\sqrt{S_{i}\times t_{i}}$ (S: number of reads for all 2bRAD markers mapped to species i in the sample; t: number of all 2bRAD markers mapped to species i in the sample). Screening of species with G score above the threshold of 5 as candidate species. Subsequently, the relative abundance of each species in the sample was calculated using the following formula: Relative abundance _species i_ = $\frac{S_{i}/T_{i}}{\sum_{i=1}^{n}S_{i}/T_{i}}$ (S: number of reads for all 2bRAD markers mapped to species i in the sample; T: number of all 2bRAD markers for species i in the database).

### 2.5. Statistical analysis

R software was used to conduct statistical analysis. The alpha diversity of groups C and B were compared using the paired Wilcoxon test for Chao1 (species number and richness), Shannon index (species richness and evenness) and Simpson index (species diversity). The differences in beta diversity between groups C and B were statistically compared using PCoA analysis for Binary Jaccard distance, Bray–Curtis distance, and Euclidean distance. Moreover, differential species in the two groups were analyzed using Kruskal Wallis. The results were regarded as statistically significant based on *p*-value <0.05.

## 3. Results

### 3.1. Ovarian microbial diversity of cancer and benign tissues

**Figure 1:**
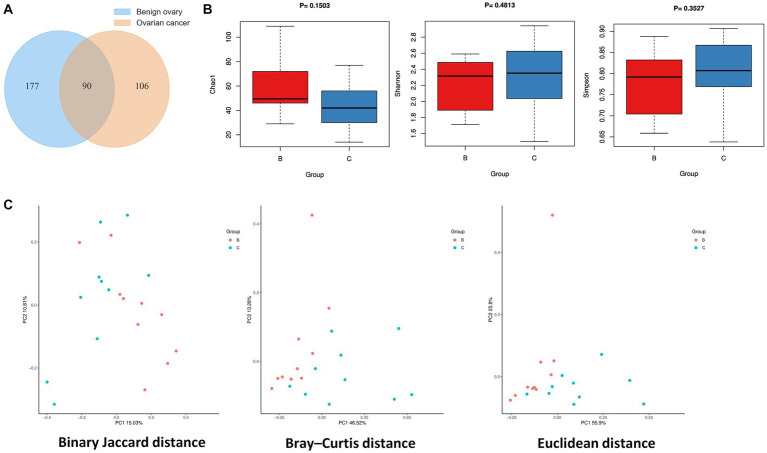
Ovarian microbial diversity of cancer and benign tissues. **(A)** Venn diagram showing the overlap of microbial species between ovarian cancer and benign ovarian tissues. **(B)** Comparison of differences in alpha diversity between group B and group C. **(C)** Comparison of differences in beta diversity between group B and group C based on 2D-PCoA. Each point corresponds to a sample, where the red point represents group B and the blue point represents group C.

The statistics of data volume changes during sequencing quality control are shown in Supplementary Table S3, including raw reads, enzyme reads, and clean reads. A total of 373 microbial species were identified from ovarian cancer and benign ovarian tissues, and Venn diagram showed 90 species overlapped between the two groups (Figure 1A). There are 106 special species in ovarian cancer tissues, and 177 in the benign ovarian tissues. Next, we analyzed the microbial alpha diversity. As shown in Figure 1B, Chao1, Shannon index, and Simpson index were not significantly different in group C and group B. Nevertheless, there was a tendency for the number of species to be higher in group B than in group C, while species diversity was lower than in group C. Additionally, we found some differences in the microbial composition of group B and group C by PCoA analysis (Figure 1C).

### 3.2. Ovarian microbial community composition

**Figure 2:**
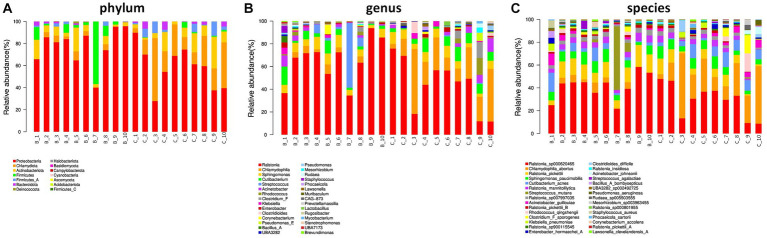
Ovarian microbial community composition. **(A)** The relative abundance of bacterial phylum in the two groups. **(B,C)** The top 30 most abundant bacterial genus **(B)** and species **(C)** in the two groups.

In terms of phylum level, proteobacteria, chlamydiota, and actinobacteriota were dominant phylum in the two groups. Interestingly, firmicutes was major in benign ovarian tissues (Figure 2A). At the genus level, *ralstonia*, *chlamydophila*, and *sphingomonas* were predominate genus in both the two groups (Figure 2B). In addition, the top five most abundant species were *ralstonia_sp000620465*, *chlamydophila_abortus*, *ralstonia_pickettii*, *sphingomonas_paucimobilis*, and *cutibacterium_acnes* in both groups (Figure 2C).

### 3.3. Differential abundance of microbial species between ovarian cancer and benign ovarian tissues

**Figure 3:**
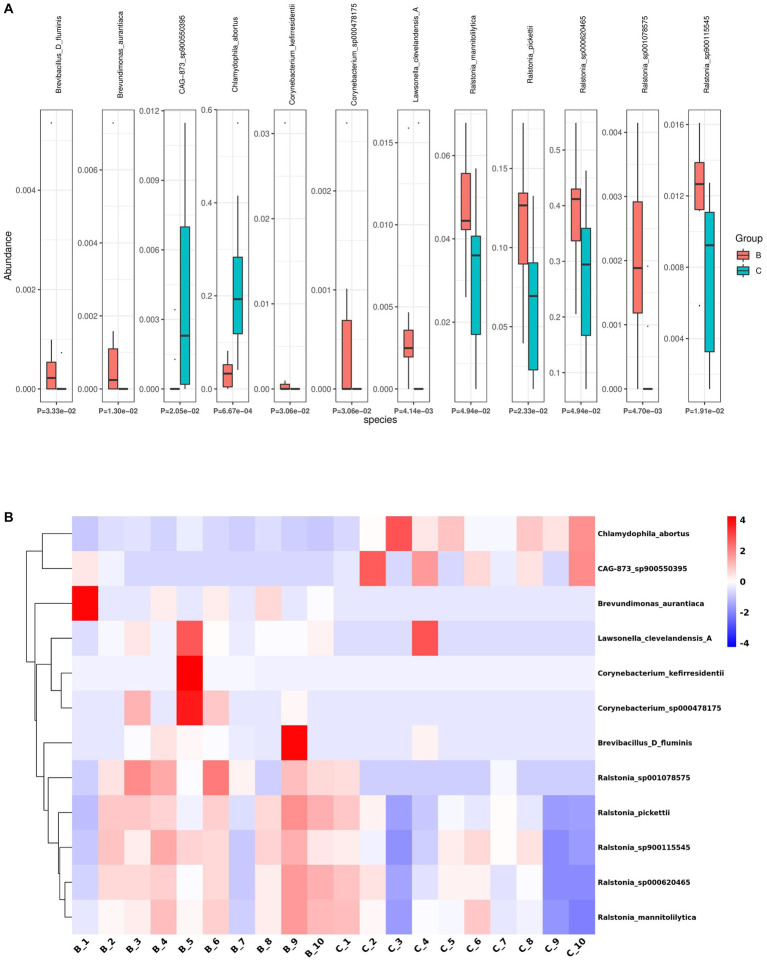
Differential abundance of microbial species between ovarian cancer and benign ovarian tissues. **(A,B)** The boxplot **(A)** and heatmap **(B)** show the differences in bacterial species in group B and group C by Kruskal Wallis analysis.

**Figure 4:**
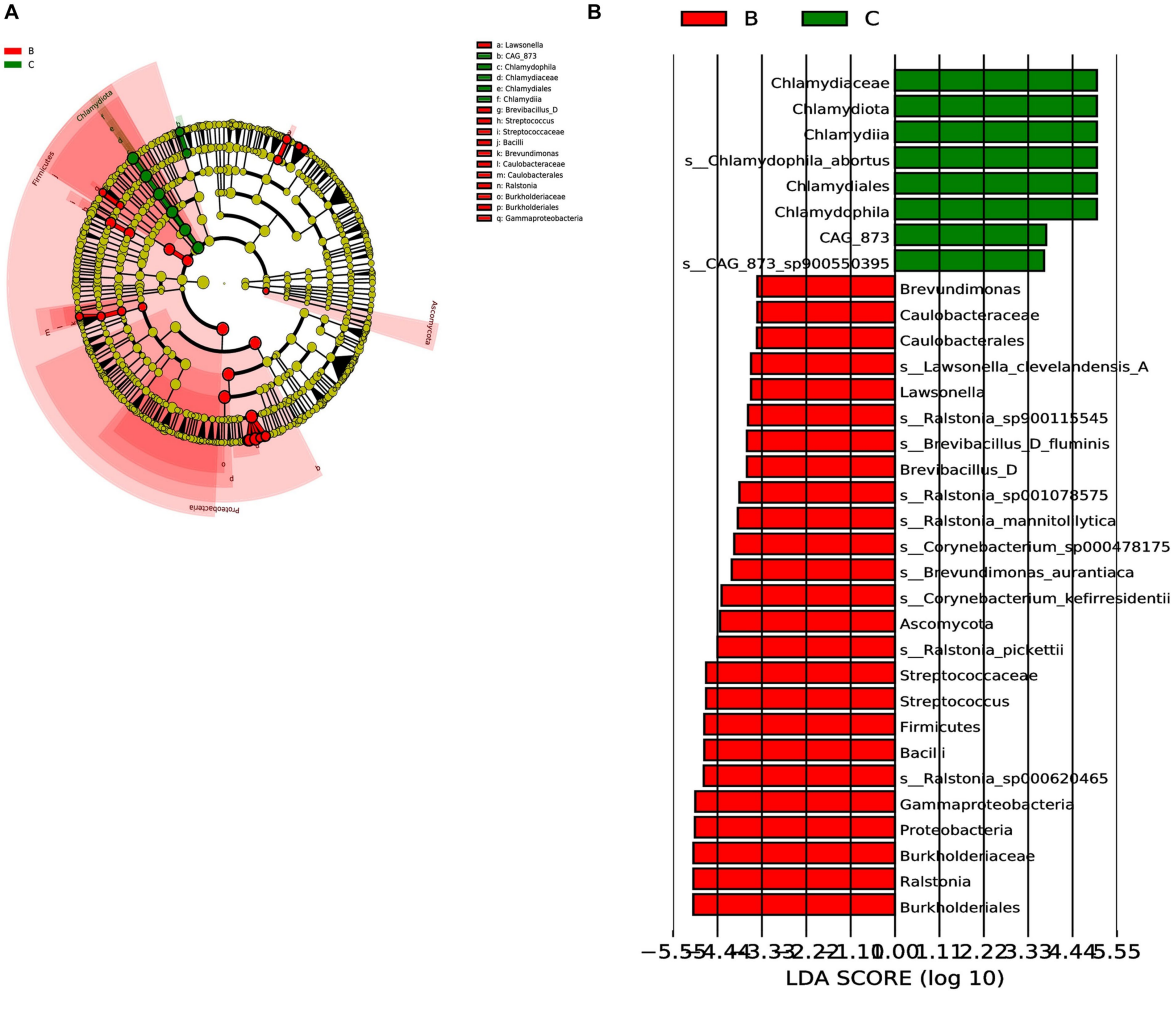
LEfSe was performed to identify diferential abundances of bacterial taxa between the two groups. **(A)** The Cladogram represents the taxonomic hierarchical structure biomarkers identified by LEfSe. **(B)** The histogram of LDA score showed significantly different biomarkers between the two groups.

Next, we analyzed the differences in microbial composition between the cancer group (C) and the control group (B) at the phylum, genus, and species levels (Table 1). The results indicated that Chlamydiota was significantly higher in ovarian cancer tissues than in benign ovarian tissues at the phylum level. At the genus level, ovarian cancer tissues had significantly higher abundance of *Chlamydophila* and *CAG-873* and significantly lower abundance of *Lawsonella*, *Brevundimonas*, *Brevibacillus_D*, *Streptococcus*, and *Ralstonia*. At the species level, we found that *Chlamydophila_abortus* and *CAG-873_sp900550395* were significantly enriched in ovarian cancer, while *Lawsonella_clevelandensis_A*, *Ralstonia_sp001078575*, *Brevundimonas_aurantiaca*, *Ralstonia_sp900115545*, *Ralstonia_pickettii*, *Corynebacterium_kefirresidentii*, *Corynebacterium_sp000478175*, *Brevibacillus_D_fluminis*, *Ralstonia_sp000620465*, and *Ralstonia_mannitolilytica* were more frequently identified in benign ovarian tissues (Figures 3A,B). Furthermore, we also identified seven fungal species in ovarian tissue, of which *malassezia_globosa* was slightly more abundant in ovarian cancer tissues than in benign ovarian tissues, but there was no significant difference (Supplementary Figure S1). We also analyzed the composition of the two groups of differential species by LEfSe. LEfSe identified 33 discriminative features (LDA score ≥ 2.0) with significant differences in relative abundance between group B and group C (Figures 4A,B).

**Table 1:** Microbial composition between ovarian cancer and benign ovarian tissues at the phylum, genus, and species levels.

Taxa		Average relative abundance		
		Benign ovary	Ovarian cancer	*p*-value
phylum	Chlamydiota	0.0319	0.2331	0.0007
	Firmicutes	0.0943	0.0092	0.0126
	Ascomycota	0.0001	0.000	0.0130
	Proteobacteria	0.7729	0.5826	0.0284
Genus	Chlamydophila	0.0319	0.2331	0.0007
	Lawsonella	0.0037	0.0016	0.0041
	Brevundimonas	0.0026	0.0003	0.0049
	CAG-873	0.0005	0.0044	0.0205
	Brevibacillus_D	0.0008	0.0001	0.0333
	Streptococcus	0.0759	0.0021	0.0406
	Ralstonia	0.6333	0.4230	0.0494
Species	Chlamydophila_abortus	0.0319	0.2331	0.0007
	Lawsonella_clevelandensis_A	0.0037	0.0016	0.0041
	Ralstonia_sp001078575	0.0020	0.0003	0.0047
	Brevundimonas_aurantiaca	0.0011	0.0000	0.013
	Ralstonia_sp900115545	0.0119	0.0076	0.0191
	CAG-873_sp900550395	0.0005	0.0038	0.0205
	Ralstonia_pickettii	0.1158	0.0633	0.0233
	Corynebacterium_kefirresidentii	0.0033	0.0000	0.0306
	Corynebacterium_sp000478175	0.0005	0.0000	0.0306
	Brevibacillus_D_fluminis	0.0008	0.0001	0.0333
	Ralstonia_sp000620465	0.3867	0.2706	0.0494
	Ralstonia_mannitolilytica	0.0468	0.0321	0.0494

## 4. Discussion

Ovarian cancer is a highly heterogeneous (including histological and molecular biology) cancer that has been shown to probably originate in the fallopian tubes (Vaughan et al., 2011; Venkatesan, 2017). Ovarian cancer, one of the deadliest types of gynecological cancer, is usually not diagnosed until advanced stages or until it has metastasized to the peritoneum and omentum, while approximately only 20% of cases are detected in the early stages (Matulonis et al., 2016). Research has shown that environmental, genetic and microbiological factors are common major factors in the development of ovarian cancer (Dhingra et al., 2022). About only 5–10% of ovarian cancer patients are hereditary (Hollis and Gourley, 2016). Therefore, it is very likely that microorganisms have an impact on the occurrence and development of ovarian cancer.

Previous studies have shown that intestinal microbial dysbiosis is closely related to the progression of ovarian cancer and platinum sensitivity (Xu et al., 2019; D'Amico et al., 2021). It has been reported that the perturbation of intestinal microbiota could promote the growth of ovarian cancer tumors and confer cisplatin resistance (Chambers et al., 2022). In healthy female the reproductive system is dominated by *Lactobacillus*, preventing the invasion and migration of pathogenic microorganisms (Xu et al., 2020). Meanwhile, a 2017 study showed that a unique set of viral, bacterial, fungal and parasitic signatures were detected in ovarian cancer samples using pan-pathogen array (PathoChip), which may contribute to the carcinogenic process (Banerjee et al., 2017). Zhou et al. (2019) found a significant decrease in microbial diversity and abundance in ovarian cancer tissues and a significantly higher proportion of *Proteobacteria*/*Firmicutes* phylum in ovarian cancer. Additionally, Wang et al. showed an increase *Aquificae* and *Planctomycetes* and a decrease *Crenarchaeota* in ovarian cancer tissues (Wang et al., 2020). Interestingly, in addition to ovarian cancer, endometriosis is also closely related to gut microbial composition, which may lay the foundation for the treatment of endometriosis patients with microbial characteristics with probiotics before surgery (Iavarone et al., 2023). In summary, although these studies have shown the presence of microbial communities in ovarian cancer tissues by 16S rRNA sequencing, they were all based on detection at the genus level. Herein, we aim to characterize the microbiome of ovarian cancer tissues and explore whether there are microbial differences between benign ovarian and ovarian cancer tissues using 2bRAD-M, a new sequencing technology that can characterize the microbiome of low biomass samples with high precision and low cost at the species level.

In this study, alpha diversity indicated that the microbial species richness of benign ovarian tissues was slightly higher than that of ovarian cancer tissues, which was consistent with previous studies (Wang et al., 2020; Han et al., 2023). However, there was no significant difference in microbial abundance and diversity between group C and group B, which we hypothesized might be due to the use of ovarian tissue from patients with ovarian cysts as the control group, since we were unable to collect ovarian tissue from healthy women. For beta diversity, we observed some differences in the microbial composition of benign ovarian tissues and ovarian cancer tissues, especially Bray-Curtis and Euclidean distance showed a relatively obvious distinction between the two groups. Therefore, we speculate that certain microorganisms in ovarian cancer tissues may serve as biomarkers for ovarian cancer diagnosis. A previous study suggested that a significant reduction of *lactococcu*s in ovarian cancer tissue might be a potential biomarker for the disease (Han et al., 2023). Previous studies have shown that exosomal miRNAs from liquid biopsy (such as miR-191 and miR-195, etc.) can be used as biomarkers for early diagnosis of endometriosis (Ronsini et al., 2023). Similarly, ovarian cancer tissue microbes can also secrete extracellular vesicles, so liquid biopsy may also serve as a means of early diagnosis of ovarian cancer.

The abundance profiles indicated that the dominant bacteria were similar in both the two groups at different taxonomic levels. More importantly, differential abundance analysis revealed that *Chlamydophila* was significantly more abundant in ovarian cancer tissues than in benign ovarian tissues. Early evidence suggests that the presence of *Mycoplasma* and *Chlamydia* in the most ovarian cancer samples (Carvalho and Carvalho, 2008; Shanmughapriya et al., 2012; Wahid et al., 2022). Moreover, previous studies have implicated *Chlamydia trachomatis* in the development of cervical and ovarian cancers, and *Chlamydia* infection promotes host DNA damage and proliferation, but impairs DNA damage repair (Chumduri et al., 2013). A recent study showed that *Chlamydia trachomatis* infection could increase the overall risk of ovarian cancer (Hosseininasab-Nodoushan et al., 2022). In addition, *CAG-873* was also significantly higher in ovarian cancer tissues than in benign ovarian tissues. *CAG-873* has been reported to be associated with acute graft-versus-host disease (Bowerman et al., 2020), however, its role in ovarian cancer has not been reported and needs to be further explored. Notably, *Ralstonia* was enriched in the ovarian cyst tissues. It is reported that the presence of *Ralstonia* spp. was associated with acute myeloid leukemia (Robinson et al., 2017). However, whether there is a correlation between *Ralstonia* and ovarian disease progression remains unclear and needs to be further explored. Interestingly, we found traces of fungi in ovarian samples, especially *malassezia_globosa* expressed in both cancerous and benign ovarian tissues. Previous studies suggest *malassezia_globosa* plays a key tumor-promoting role in the development of pancreatic ductal adenocarcinoma (Bellotti et al., 2021) and gastric cancer (Zhang et al., 2022). However, there are no reports on the fungus of ovarian cancer, which needs to be further explored.

However, there are several limitations to our study. First, for ethical reasons, we could not collect ovarian tissue from healthy women as a control group. Thus, we could only use non-cancerous ovarian tissues from patients with ovarian cysts as a control. Second our sample size was small, which limited the comparison between ovarian cancer subtypes and may have affected the accuracy of the results. A larger study population is needed for further investigation. In addition, our research was only descriptive and did not directly examine the function of ovarian cancer microbes, so it is difficult to determine the causal relationship between microorganism and ovarian cancer. Finally, we did not delve deeper into the differences in the microbial composition of different stages of ovarian cancer. As we all know, open surgery is currently the most common method for staging and treatment of ovarian cancer. However, minimally invasive surgery with fewer complications is also used for staging ovarian cancer (Ronsini et al., 2023). Therefore, the study of microbial composition in different stages of ovarian cancer may provide assistance for surgical staging.

In summary, our study is the first to utilize 2bRAD-M, a new microbial sequencing technology that can accurately characterize the low-biomass ovarian cancer microbiome at the species level. Our research demonstrates that the microbiome composition differed between cancerous and benign ovaries, with 12 species showing significant differences in relative abundance between the two groups. Notably, our findings differ from previous results using 16S rRNA to detect ovarian cancer microorganisms. Overall, our study provides further insight into the ovarian cancer microbiome. In addition, we identified microbial species differential between ovarian cancer and benign ovarian tissues, which may help to explain the carcinogenesis process of ovarian cancer and provide new ideas for clinical diagnostic biomarkers, microbial therapy and microbial prognostic targets. However, further research is needed to gain insight into the relationship between ovarian cancer and microbes.

## Data availability statement

The original contributions presented in the study are publicly available. This data can be found here: https://www.ncbi.nlm.nih.gov/sra/PRJNA1005427.

## Ethics statement

The studies involving humans were approved by Ethical Review Committee of Nanjing First Hospital. The studies were conducted in accordance with the local legislation and institutional requirements. The participants provided their written informed consent to participate in this study.

## Author contributions

XW, TW, ShuZ and SS designed and coordinated the project. XW performed the experiments and drafted the manuscript. YZ, XC, CP, and ShiZ helped perform the experiments and analyzed the data. ShuZ helped collect tissue sample. All authors contributed to the article and approved the submitted version.

## Funding

This work is supported by grants from National Natural Science Foundation of China (82072648, 82273011, 82173205), the Natural Science Foundation of Jiangsu Province (BK20211508), the Fundamental Research Funds for the Central Universities (021414380500).

## Conflict of interest

The authors declare that the research was conducted in the absence of any commercial or financial relationships that could be construed as a potential conflict of interest.

## Publisher’s note

All claims expressed in this article are solely those of the authors and do not necessarily represent those of their affiliated organizations, or those of the publisher, the editors and the reviewers. Any product that may be evaluated in this article, or claim that may be made by its manufacturer, is not guaranteed or endorsed by the publisher.
