# Supplementary material for: 2bRAD-M reveals the difference in microbial distribution between cancerous and benign ovarian tissues
Source: Front Microbiol. 2023 Aug 24;14:1231354. doi: 10.3389/fmicb.2023.1231354 (PMC10484612; doi:10.3389/fmicb.2023.1231354)
Supplement: Supplementary file 1 [file Data_Sheet_1.docx]

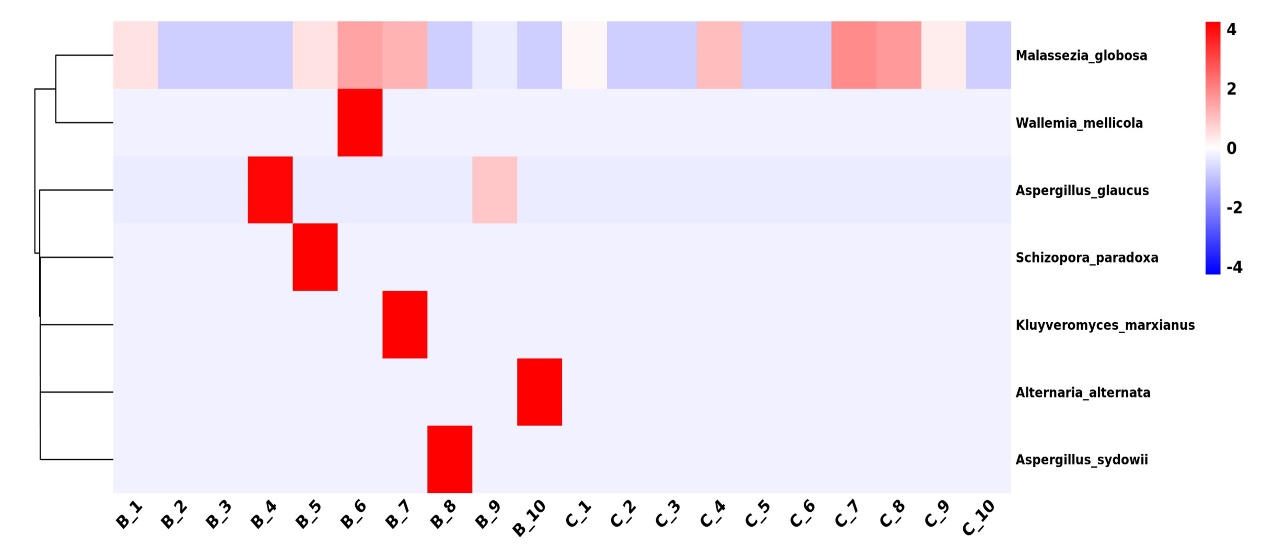
**Figure S1**

**Figure S1.** The heatmap show the fungal species in the two groups.

**Table S1. Clinical data for patients of ovarian cancer and ovarian cyst**

| **Ovarian cancer (C)** | | | | | | **Ovarian cyst/Benign ovary (B)** | | | |
| --- | --- | --- | --- | --- | --- | --- | --- | --- | --- |
|  | **Age** | **Histology** | **FIGO stage** | **Tumor grade** | **Metastasis** |  | **Age** | **Type** | **Location** |
| C_1 | 47 | Serous | IV | G3 | Yes | B_1 | 35 | Benign cyst adenoma | Ovarian |
| C_2 | 50 | Mucinous | IIIc | G3 | Yes | B_2 | 44 | Functional cysts | Ovarian |
| C_3 | 60 | Serous | IIb | G2 | Yes | B_3 | 47 | Functional cysts | Ovarian |
| C_4 | 49 | Serous | IIIc | G3 | Yes | B_4 | 55 | Benign cyst adenoma | Ovarian |
| C_5 | 56 | Serous | IV | G3 | Yes | B_5 | 48 | Benign cyst adenoma | Ovarian |
| C_6 | 43 | Serous | IV | G3 | Yes | B_6 | 42 | Benign cyst adenoma | Ovarian |
| C_7 | 62 | Clear cell | Ic | G2 | No | B_7 | 37 | Functional cysts | Ovarian |
| C_8 | 72 | Serous | IV | G3 | Yes | B_8 | 46 | Benign cyst adenoma | Ovarian |
| C_9 | 40 | Serous | IIIc | G3 | Yes | B_9 | 58 | Benign cyst adenoma | Ovarian |
| C_10 | 57 | Mucinous | IV | G3 | Yes | B_10 | 62 | Functional cysts | Ovarian |

**Table S2. Adaptors and primers used for 2bRAD-M (5’- 3’)**

| **Adaptors** | **Sequence (5’ - 3’)** |
| --- | --- |
| Adap-1 sense | ACACTCTTTCCCTACACGACGCTCTTCCGATCTNN |
| Adap-2 sense | GTGACTGGAGTTCAGACGTGTGCTCTTCCGATCTNN |
| Adap antisense | AGATCGGAAGAGC |
| **Primers** | **Sequence (5’ - 3’)** |
| Primer1 | ACACTCTTTCCCTACACGACGCT |
| Primer2 | GTGACTGGAGTTCAGACGTGTGCT |
| 5UDI Primer | AATGATACGGCGACCACCGAGATCTACACXXXXXXXXACACTCTTTCCCTACACGACGCTCTTCCGATCT |
| 7UDI Primer | CAAGCAGAAGACGGCATACGAGATXXXXXXXXGTGACTGGAGTTCAGACGTGTGCTCTTCCGATCT |

**Table S3. Change in data volume during quality control: raw reads, enzyme reads, and clean reads and percentage of clean reads**

| **Sample** | **Raw reads** | **Enzyme reads** | **Clean reads** | **Percent** |
| --- | --- | --- | --- | --- |
| B_1 | 7720507 | 6527211 | 6158001 | 79.76% |
| B_2 | 12747572 | 11288951 | 10635816 | 83.43% |
| B_3 | 11781846 | 11044031 | 10387068 | 88.16% |
| B_4 | 12947739 | 11462869 | 10804366 | 83.45% |
| B_5 | 12255277 | 10811022 | 10173729 | 83.02% |
| B_6 | 8901165 | 8555399 | 8097984 | 90.98% |
| B_7 | 8958047 | 7633936 | 7211801 | 80.51% |
| B_8 | 4865241 | 4174453 | 3930977 | 80.80% |
| B_9 | 8749157 | 7692115 | 7293303 | 83.36% |
| B_10 | 6365623 | 6025491 | 5701145 | 89.56% |
| C_1 | 12499892 | 11113805 | 10456718 | 83.65% |
| C_2 | 7000437 | 6225324 | 5870118 | 83.85% |
| C_3 | 7609316 | 7076184 | 6655973 | 87.47% |
| C_4 | 6614824 | 6140602 | 5767204 | 87.19% |
| C_5 | 4938219 | 4359326 | 4083439 | 82.69% |
| C_6 | 6996532 | 6248532 | 5841945 | 83.50% |
| C_7 | 7139427 | 6782562 | 6340029 | 88.80% |
| C_8 | 8816328 | 8497315 | 7965392 | 90.35% |
| C_9 | 10035099 | 7533618 | 7047432 | 70.23% |
| C_10 | 7131004 | 6847245 | 6446441 | 90.40% |
